# Systematic review and validity assessment of methods used in discrete choice experiments of primary healthcare professionals

**DOI:** 10.1186/s13561-020-00295-8

**Published:** 2020-12-09

**Authors:** Gregory Merlo, Mieke van Driel, Lisa Hall

**Affiliations:** 1 Primary Care Clinical Unit, Faculty of Medicine, University of Queensland, Level 8 Health Sciences Building, Building 16/910, Royal Brisbane & Women’s Hospital, Brisbane, QLD 4029 Australia; 2 School of Public Health, Faculty of Medicine, University of Queensland, Brisbane, Australia

**Keywords:** Discrete choice experiment, Systematic review, Primary care, General practice, Family practice

## Abstract

**Introduction:**

Discrete choice experiments (DCEs) have been used to measure patient and healthcare professionals preferences in a range of settings internationally. Using DCEs in primary care is valuable for determining how to improve rational shared decision making. The purpose of this systematic review is to assess the validity of the methods used for DCEs assessing the decision making of healthcare professionals in primary care.

**Main body:**

A systematic search was conducted to identify articles with original data from a discrete choice experiment where the population was primary healthcare professionals. All publication dates from database inception to 29th February 2020 were included. A data extraction and validity assessment template based on guidelines was used. After screening, 34 studies met the eligibility criteria and were included in the systematic review. The sample sizes of the DCEs ranged from 10 to 3727. The published DCEs often provided insufficient detail about the process of determining the attributes and levels. The majority of the studies did not involve primary care healthcare professionals outside of the research team in attribute identification and selection. Less than 80% of the DCEs were piloted and few papers investigated internal or external validity.

**Conclusions:**

For findings to translate into improvements in rational shared decision making in primary care DCEs need to be internally and externally valid and the findings need to be able to be communicated to stakeholders in a way that is understandable and relevant.

## Introduction

Discrete choice experiments (DCEs) have been widely used in economics and marketing to assess how much people value the attributes of a good or service [1]. It is a method based on Lancaster’s theory, which is that a good or a service can be described by its attributes and that a person’s preferences for a good or service depends on their preferences for the attributes of that good or service. For example, a person’s preference for a house will depend on the attributes of that house—e.g. location, number of rooms, or the condition of the interior. A discrete choice experiment is conducted to measure the preferences for these attributes, particularly when it is not possible to measure through revealed behaviour.

A DCE consists of choice tasks, where a respondent is asked, as a hypothetical scenario, to choose between two or more discrete alternatives (e.g. House A or House B) as the attributes that define those alternatives are varied. The choices made are then analysed to measure how much the respondents value these attributes. The advantage of using a DCE over other preference elicitation techniques is that it forces respondents to make explicit trade-offs.

DCE methodology has evolved over time. There has been increased sophistication in use of statistical methods and experimental designs, such as measurement of interaction effects and use of Bayesian efficient designs [2, 3]. There has been increased emphasis on using valid research methods to inform choice task design in terms of defining levels, attributes, and scenarios [4]. Greater methodological sophistication has coincided with the development of guidelines for conducting and reporting economic evaluations [5, 6] and a focus on improving the internal and external validity of DCEs [2, 7].

Over the past decades DCEs have been used to measure patient and healthcare provider preferences in a wide range of settings internationally [2]. DCEs have been used in health technology assessment, workforce planning, and broader policy making [1, 8]. Systematic reviews of DCEs have measured healthcare professionals’ preferences for where they would like to work [3], patient and physician preferences for specific conditions or treatments [9, 10], disease specific quality of life measurements [11], decision making preferences for specific countries or geographical areas [12, 13], and methodological issues such as development of attributes [14] or assessing external validation [7].

DCEs are a valuable tool for determining how to improve rational shared decision making in primary care. Seemingly minor decisions made in primary care can have large and long-term effects on a person’s health and their experience with the healthcare system. These decisions involve trade-offs—choosing a treatment may involve a trade-off between effectiveness, adverse effects, convenience, and cost. A role of the physician in shared decision making is to help patients navigate these trade-offs. However, both the patient and clinician are subject to biases that may result in non-rational decision making [15]. The advantage of using a DCE is that it asks individuals to explicitly make trade-offs that would not otherwise be able to observe—providing a better understanding of the factors that influence the decision making of health professionals.

A previously published systematic review demonstrated the role of DCEs in understanding patient preferences in primary care [16]. The review revealed a diversity of healthcare decisions where DCEs have been used to measure patient preferences and describe common factors that influence those decisions. The review also identified methodological challenges with conducting DCEs in primary care. However, no systematic review has investigated the use of DCEs to explore the decisions of primary care healthcare professionals.

The purpose of our systematic review is to assess the methods used in DCEs that assess the decision making of *healthcare professionals* in primary care [17], and the validity of the reported studies .

## Methods

Studies on the use of DCEs of primary healthcare professionals were identified, selected, and analysed in accordance with the Preferred Reporting Items for Systematic Reviews and Meta-Analyses (PRISMA) [18]. A systematic search strategy (Supplement 1) was used to search Embase, CINAHL, and ECONLIT (30th July, 2019). Citations identified through database searching were imported into Endnote and duplicates were removed. Studies were screened for eligibility based on the title and abstract, followed by full-text screening by two researchers.

To be included the studies had to report original data from a DCE. The participants had to include primary healthcare professionals or general practitioners. Studies that did not report outcomes specific for primary healthcare professionals or general practitioners were excluded. All publication dates from database inception to 29th February 2020 were included. Non-English language articles were excluded. Articles were excluded if they were conference abstracts or did not report original research, such as letters to the editor, editorials, and opinion articles.

Studies meeting the review eligibility criteria after full-text screening were included in the review. Two researchers extracted, reviewed and summarised data according to a data extraction template, which included a validity assessment checklist. A third researcher adjudicated in cases of differences or discrepancies. The findings were summarised using a descriptive synthesis. Articles were grouped by areas of application of the DCEs. The data extraction template and validity assessment checklist was adapted from Mandeville et al. [3].

## Results

**Fig. 1:**
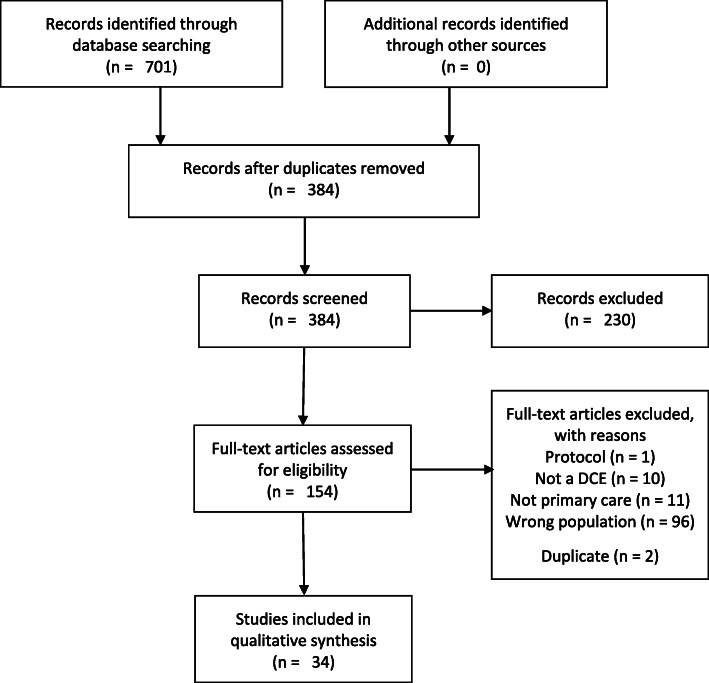
PRISMA flow diagram of study selection [18]

The search of the databases identified 701 records. After screening, 34 studies met the eligibility criteria and were included in the systematic review (See Fig. 1). The included DCEs have been published from 2000 to 2020. The majority (82.4%) of the studies were published between 2010 and 2020. For full description of the studies, including subject of the DCE, populations, and attributes assessed see Supplement 2.

### Data collection

Table 1 presents the sample and survey administration details for the included studies. More than half (*N* = 21, 61.8%) of the DCEs included only primary healthcare professionals. Of the studies that included other populations as well as health professionals, five (14.7%) included patients, ten (29.4%) included other health professionals such as specialists, and one (2.9%) included policymakers. Twenty (58.8%) of the DCEs were administered as online surveys, nine (26.5%) were postal surveys, and two (5.9%) were administered in face-to-face interviews. The sample sizes of the DCEs ranged from 10 to 3727, with a median of 294. Twenty-two (64.7%) of the studies reported a response rate. The median response rate was 42%. In over 90% of the included studies the target population was appropriate and the sampling frame was representative of the target population. In the majority (73.5%) of the studies piloting of the DCE instrument was undertaken before the survey was conducted.

**Table 1:** Data collection for included DCEs

Item	Category	Number of studies	%
Sample	Primary healthcare professionals only	21	61.8%
	with other health professionals	8	23.5%
	with patients	3	8.8%
	With other health professionals and patients	1	2.9%
	with patients and policymakers	1	2.9%
Administration of survey	Interview	2	5.9%
	Postal survey	9	26.5%
	Online survey	20	58.8%
	Both online and postal	3	8.8%
Sample size primary healthcare professionals	< 50	4	11.8%
	50–200	7	20.6%
	200–500	13	38.2%
	> 500	10	29.4%
Response rate	< 30%	9	26.5%
	30–60%	9	26.5%
	> 60%	4	11.8%
	Not reported	12	35.3%
Piloting	Yes	25	73.5%
Response incentive	Yes	4	11.8%

### Purpose of DCE

Fourteen of the studies assessed healthcare professional decision making about therapy and disease management [19–32], twelve assessed preferences for organisation characteristics of primary care practices [33–44], seven assessed the relative influence of implementation and knowledge translation strategies [45–50], and two assessed preferences for information and communication technologies [51, 52].

### Choice task design

The most common methods for identifying and labelling attributes and levels were literature review (96.9%), followed by interviews and expert opinion (62.5%), and focus groups (25.0%). Twenty-three (67.6%) of the studies used more than one method to identify and label the attributes and levels. The number of attributes ranged from four to ten, with 70.6% having between five and seven attributes. For the majority of the studies there was no conceptual overlap between attributes (82.4%) and the attributes were uni-dimensional (76.5%), as recommended by Lancsar and Louviere [6].

The most common attribute used in the DCEs assessing physician preferences for therapy and disease was cost [19, 20, 22, 28, 29, 31], including both cost of the intervention [20, 22, 28, 29, 31] and direct cost to the patient [19, 20, 29, 31]. Other frequently used attributes included patient preferences [19–21, 31, 38, 53], effectiveness of the intervention [19, 20, 29–31], health professional’s experience with either the patient or the intervention [20, 23, 29, 31, 53], and disease characteristics such as level of functional impairment or duration [21, 24, 25, 32, 53].

Within the studies assessing physician choice of where to work the most frequently used attributes were income [34, 36, 37, 39–41, 43, 44], working hours [34–36, 39, 41, 44], opportunities for professional development [34–36, 41, 43, 44], size of practice [34, 35, 39, 40, 42], and characteristics of the patient population [33, 34, 41, 43], such as list size and level of social disadvantage in the community.

For the DCEs exploring implementation and knowledge translation strategies, the most frequently used attributes were those related to mode and design of knowledge translation [45–49], the presence of financial incentives [45, 47, 49, 50], and feedback [45, 47, 48].

Generic choice set labelling, where the choice sets are unlabelled alternatives, was more common (*n* = 25; 73.5%), than labelled choice sets (*n* = 9; 26.5%), where the labels for the choices provided information regarding the alternatives.

Most of the DCE instruments did not provide an alternative option (n = 25; 73.5%) and instead had a forced choice where one of the discrete choice options had to be chosen. For those that did provide an alternative option, the alternative was defined as having the option to either remain in the status quo (*n* = 2; 5.9%), to opt-out and pick neither alternative (*n* = 3; 8.8%), or to remain undecided (n = 3; 8.8%). For example, a DCE of health professional’s decision to choose which practice they would work at had a status quo option where they could continue to work at their current practice rather than one of the two choice sets provided describing alternative GP practices.

### Experimental design

Most of the studies used an experimental design to limit the number of choices the respondents had to make, although one study used a full factorial design (see Table 2). Nineteen (55.9%) of the studies that used an experimental design were fractional factorial designs, eleven (32.4%) were efficient designs, and three studies (8.8%) did not report type of design. The majority (79.4%) of the studies used blocking, where each respondent is randomised to only answer a subset of the choice tasks [54].

**Table 2:** Experimental design

Criteria	Category	Number of studies	%
Type of design	Full factorial	1	2.9%
	Fractional factorial	19	55.9%
	“Efficient design”	6	17.6%
	Bayesian efficient design	5	14.7%
	Not reported	3	8.8%
Generation of choice sets	Software	22	64.7%
	Manual	2	5.9%
	Not reported	9	26.5%
Design aims	Level balance	7	14.7%
	Minimal overlap	3	8.8%
	Orthogonality	14	41.2%
	Other	1	2.9%
Design efficiency	D-Efficient	16	47.1%
	Unclear or not reported	18	52.9%
Blocked design	Blocked	27	79.4%
	No blocking	3	8.8%
	Unclear or not reported	4	11.8%

### Analysis procedure and statistical tests

The most common estimation model used for statistical analysis was the mixed logit (*N* = 15, 44.1%), followed by the conditional or multinomial logit model (*N* = 13, 38.2%; see Table 3). Twenty (58.8%) of the studies explicitly discussed goodness of fit.. Level coding was discussed in 21 (38.2%) of the studies—of those 9 used effects coding and 12 used dummy coding. Interaction effects were measured in 17 (50.0%) of the studies and 19 (55.9%) reported a measure of marginal rate of substitution, such as willingness to pay, willingness to wait, or willingness to accept a social cost. Sociodemographics and other covariates were included in the analysis for 13 (38.2%) of the studies. Out of 208 attributes that were assessed for impact on respondent choice in the DCEs, 182 (87.5%) were significant. None of the attributes were dominant, which is when an attribute is so highly preferred that an individual is not willing to make trade-offs with other attributes [55].

**Table 3:** Analysis procedure and statistical tests

Item	Category	Number of studies	%
Level coding discussed	Yes	21	61.8%
	No	13	38.2%
Goodness of fit considered	Yes	20	58.8%
	No	8	23.5%
	Unclear or not reported	6	17.6%
Internal or external validity investigated	Yes	10	29.4%
	No	5	14.7%
	Unclear or not reported	19	55.9%
Estimation model	Conditional or multinomial logit	13	38.2%
	Random effects probit	3	8.8%
	Latent-class analysis	2	5.9%
	Mixed logit	15	44.1%
	Random effects conditional logit	2	5.9%
	Other	2	5.9%
	Unclear or not reported	2	5.9%
Removed responses	Reasons for removal reported	6	17.6%
	Unclear or not reported	28	82.4%
Model	Akaike information criteria	3	8.8%
	Bayesian information criteria	4	11.8%
	Likelihood ratio test	11	32.4%
	Pseudo Rsquared	1	2.9%
	Other	2	5.9%
	Not reported	16	47.1%

Ten (29.4%) studies explicitly investigated internal or external validity. Examples of internal or external validity checks used in these studies included using a dominant choice task to assess internal consistency [51], detecting repetitive patterns or strait-lining in response patterns [25], rushing [25], including a duplicate question [26, 27, 44], tests of transitivity [26, 35] using follow-up questions [45], and reviewing consistency of answers [19, 35, 50]. Studies assessed external validity by comparing the DCE findings with the predicted direction of preferences [19, 37] or with findings from previously published surveys or clinical trials [25]. —for instance preferences for cheaper for effective interventions [24].

### Validity assessment

Validity assessment is presented in Table 4. The majority of studies did not provide a justification for using forced choice (70.6%). The majority of studies did not report that the experimental design was optimal or statistically efficient (67.7%). Most studies met the other validity criteria, however only two of the studies met all of the validity criteria [40, 49].

**Table 4:**
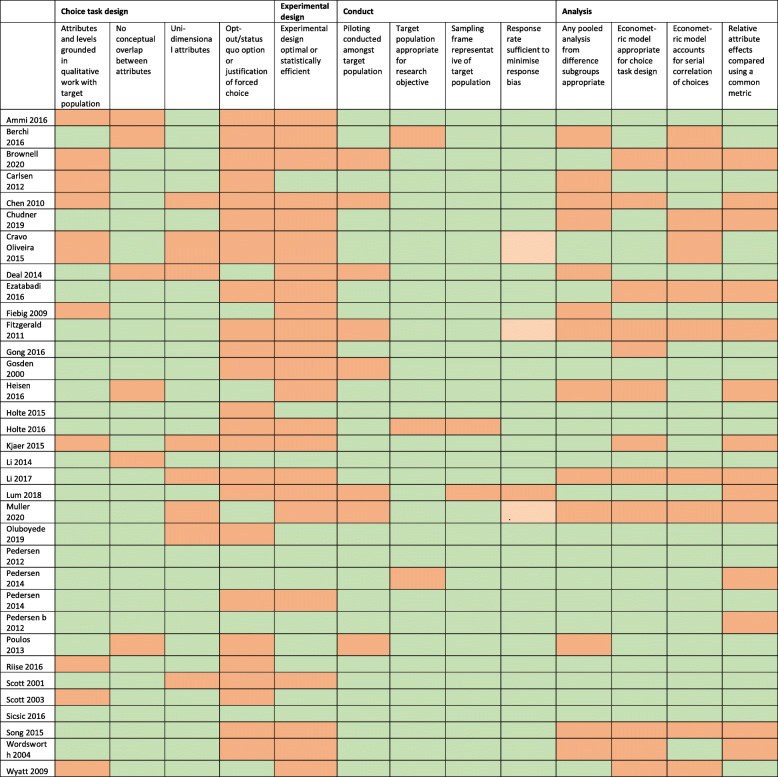
Validity assessment of included studies [3]

Red = criteria not met (no evidence or not enough evidence to justify the criteria in the text); Green = criteria met (the text sufficiently confirmed the criteria).

## Discussion

This is the first comprehensive review of DCE studies of primary healthcare professionals. Primary care is a complex and diverse environment that involves many decisions that are important to patients. The decisions made in primary care are highly individualised to the relationship between the patient and the primary care health professional. Therefore, findings from DCEs conducted in large institutions such as hospitals or that are aimed at the level of policymakers will not be generalizable [6, 16].

[3] We found only 34 studies employing DCEs to investigate health care professional decision making, most published in the last 10 years. For DCEs to be useful in helping us better understand and improve decision making in primary care they need to employ robust methods. However, we identified important limitations in the methods used in the DCEs.

The findings from the validity assessment mirrored the results of the validity assessment from Mandeville et al. [3], with a high number of studies that did not provide justification if they used forced choice, did not describe a process of developing a statistically efficient experimental design, and several studies did not describe the process of using qualitative research with the target population to develop the attributes and levels. There was little consistency between studies in the methods used to assess internal consistency—some of the studies used additional questions, others pre-specified expected preference direction in order to assess face validity, and others tested responses to determine dominance or for repetitive patterns.

The majority of the studies did not involve primary healthcare professionals outside of the research team in attribute identification and selection, which may jeopardise the relevance of the results if this would have changed the attributes selected.. A substantial proportion of the studies did not report piloting the DCE with the target population. The DCEs were mostly conducted as surveys rather than face-to-face interviews, which is appropriate for this population of healthcare professionals as they are less likely than other potential population groups to require additional assistance to understand the survey.

Only 13 (38.2%) of the studies incorporated level of professional experience in the analysis of the DCE responses. This is particularly a challenge in interpreting results of studies about workforce planning as early career and more established health professionals may have different preferences for workplace characteristics including level of ongoing training, capacity to move to a rural location, or salary expectations.

Our review was purposefully limited in scope. Only studies in primary care and general practice were included. Therefore, similar studies that did not specifically focus on these areas were excluded. Additionally, only studies published in English were included, which means potentially relevant information published in other languages could have been missed.

DCEs have the potential to inform primary care policy and practice; however, care is required to ensure that the DCEs are addressing research questions relevant to primary care practice, using appropriate methods, and have an emphasis on translating knowledge into practice. For findings to translate into improvements in rational shared decision making in primary care DCEs need to be internally and externally valid and the findings need to be able to be communicated to stakeholders in a way that is understandable and relevant. Better adherence to methodological reporting standards will be a good starting point.

## Supplementary Information

**Additional file 1.**

## Data Availability

Data sharing is not applicable to this article as no datasets were generated or analysed during the current study.

## Abbreviations

DCE: Discrete choice experiment
ISPOR: International Society for Pharmacoeconomics and Outcomes Research
PRISMA: Preferred Reporting Items for Systematic Reviews and Meta-Analyses
