# Supplementary material for: Systematic review and validity assessment of methods used in discrete choice experiments of primary healthcare professionals
Source: Health Econ Rev. 2020 Dec 9;10:39. doi: 10.1186/s13561-020-00295-8 (PMC7725112; doi:10.1186/s13561-020-00295-8)
Supplement: Supplementary file 1 — Additional file 1. [file 13561_2020_295_MOESM1_ESM.docx]

**SUPPLEMENTARY MATERIALS 1 – SEARCH STRATEGY**

| **Database** | **Search Strategy** | **Results** |
| --- | --- | --- |
| EMBASE | (('general practice'/exp OR 'general practice') OR ('general practitioner'/exp OR 'general practitioner') OR ('general practise registrar'/exp OR 'general practise registrar') OR 'general practice registrar' OR ('primary health care'/exp OR 'primary health care') OR ('primary healthcare'/exp OR 'primary healthcare') OR ('primary medical care'/exp OR 'primary medical care') OR ('primary care'/exp OR 'primary care')) AND (('discrete choice experiment'/exp OR 'discrete choice experiment') OR 'discrete choice experiments' OR ('conjoint analysis'/exp OR 'conjoint analysis') OR 'preference elicitation') | 315 |
| EBSCOhost Research Database (CINAHL, EconLit, MEDLINE) | (general practitioner or general practice or gp or family doctor or  primary care ) AND ( discrete choice experiment or conjoint analysis or preference elicitation) | 386 |

**SUPPLEMENTARY MATERIALS 2 – SUMMARY OF INCLUDED STUDIES**

| **Author Year**  **Country** | **Subject of DCE** | **Sample** | **N** | **Attributes covered** | **Key findings** |
| --- | --- | --- | --- | --- | --- |
| **Therapy and disease management** | | | | | |
| Berchi 2016[[19](#_ENREF_19)]  France | Drug therapy for osteoarthritis | General practitioners and rheumatol | 106 GPs; 82 rheumatol | 1. Pain relief* 2. Improvement in function* 3. Retardation of joint degradation 4. Annual cost to the patient* 5. Risk of moderate side effects* 6. Risk of serious side effects* 7. Degree of patient acceptance of treatment | GP treatment choices were significantly influenced by pain relief, improvement in function, annual cost, and serious side effects  Retardation of joint degradation and degree of patient acceptance of treatment did not influence GP treatment choice  GPs were willing to accept a 2.2% (95% CI 1.15%m 2.85%) increase in serious side effects or €225 (95% CI €148, €302) in annual cost to patient for 1 unit increase in function.  Younger GPs had a stronger aversion to risk of serious side effects |
| Brownell 2020[[32](#_ENREF_32)]  Australia | Urgent review of lung nodule | GPs | 152 | 1. Patient age* 2. Smoking history* 3. Respiratory symptoms* 4. Nodule speculation* 5. Nodule size 6. Radiologist recommendation | The factors associated with request for urgent review were nodule -, larger nodule size, presentation with haemoptysis or weight loss, recommendation for urgent review by the reporting radiologist, and female GP gender. There was significant heterogeneity in low risk lung nodules. |
| Carlsen 2012[[20](#_ENREF_20)]  Norway | Prescription choices | Doctors (GPs and hospital consultants) | 571 including 284 GPs and 287 hospital consultants | 1. Total costs* 2. Effectiveness* 3. Patient costs* 4. Patient preference* 5. Physician's experience* | Effectiveness of the medicine had the highest impact on choice to prescribe. Prescribing choice was also influenced by patient preferences, physician experience with the medicine, patient costs, and societal costs.  GPs value high clinical effectiveness less than hospital consultants do. They are also less concerned with patient preferences. |
| Cravo Oliveira 2015[[21](#_ENREF_21)]  Portugal | Dermatology referral and joint teleconsultations | General practitioners | 44 | 1. Dermatological presentation (nevus, melanoma, psoriasis)* 2. Waiting time 3. Distance 4. Pressure from patient | Patient need, represented by dermatological presentation, were all significantly associated with referring patient to dermatology teleconsultation.  Older GPs were significantly associated with referral. |
| Deal 2014[[22](#_ENREF_22)]  Canada | Cardiovascular disease management | Primary care physicians and patients | 144; 70 physicians, 74 patients | 1. Cost* 2. Speed of access* 3. Tracker values* 4. Nurse coordinator tasks* 5. Nurse coordinator cost* 6. Appointments* 7. Billing incentive* | Main determinants were speed of access, and monthly payments for a nurse coordinator.  Two segments of physicians were identified—one with greater preference for monthly subscription fee and tracker values; other with preference for nurse coordinator costs and billing incentives  Willingness to pay for all attributes was calculated |
| Fiebig 2009[[23](#_ENREF_23)]  Australia | Pap test | General practitioners and women | 215 GPs; 167 women | 1. Reason for consultation* 2. Screening interval* 3. Experience with patient* 4. Previous screening* 5. Patient age* 6. Patient income* 7. Payment to practice* | All of the attributes had a significant impact on choice to recommend pap test. The greatest effect was found for reason for consultation, time since last screening test, and age of patient (>70). |
| Fitzgerald 2011[[24](#_ENREF_24)]  Canada | Rheumatology referral | Primary care physicians and rheumatologists | 10 GPs; 14 specialists | 1. Independence 2. Work/role impact 3. Pain 4. Receiving corticosteroid 5. Comorbidities 6. Evidence of progressive major organ involvement | Significance of attributes not reported. An algorithm for patient priority based on weightings for each of the attributes was presented. |
| Heisen  2016[[25](#_ENREF_25)]  UK, the Netherlands, Germany, France, Spain | Medication for overactive bladder | Patients, physicians (including GPs, urologists, urogynocologists) | 318 physicians (143 GPs), 442 patients | 1. Micturition frequency* 2. Incontinence* 3. Nocturia* 4. Urgency* 5. Coping^[[1]](#footnote-1)^* 6. Dry mouth^[[2]](#footnote-2)^* 7. Constipation* 8. Increased heart rate* 9. Increased blood pressure* 10. Atrial fibrillation* | All of the attributes were significant. Physicians were most sensitive to severity of incontinence, nocturia (interference with night’s rest), and high risk of dry mouth associated with medicine |
| Li 2017[[26](#_ENREF_26)]  USA | Referral for single-proton emission computed tomography | Primary care physicians (including internal med specialists) and cardiologists | 202 primary care; 209 cardiologists | 1. Ease of referral* 2. Waiting time* 3. Pre-authorisation assistance* 4. Time to receive results* 5. Availability of conclusive statement* 6. Measurement of patient satisfaction* 7. Protocol for rapid stress test* 8. Communication* 9. Parking/accessibility* | All of the attributes were significant. Primary care physicians were most sensitive to “assistance in preauthorisation” (OR 3.02), “protocol for rapid stress test” (OR 2.64), and “availability of conclusive statement” (OR 2.44). |
| Lum 2018[[27](#_ENREF_27)]  Australia | Antibiotic prescribing | General practitioners | 23 | 1. Duration* 2. Life events* 3. Reassessment* 4. Familiarity with patient 5. Patient expectations* | Physicians were most sensitive to “duration of symptoms” and “patient expectations” |
| Oluboyede 2019[[28](#_ENREF_28)]  UK | Novel non-invasive device for diagnosis of peripheral arterial disease | Primary care clinicians | Doctors 95; Nurses 17; other 4 | 1. Devices display 2. Data integration* 3. Training* 4. Cost* 5. Power supply* 6. Portability | There was significant preference for manual integration of test results into patient records compared with automated integration; strong preference for disposable batters of power supply compared with rechargeable batteries or wired charging |
| Pedersen 2014[[29](#_ENREF_29)]  Denmark | Medication prescribing | GPs | 309 | 1. Benefit/effect* 2. Patient cost* 3. Total cost* 4. Patient attitude* 5. Experience with medicine* 6. National recommendation | Physicians were most sensitive to level of medication effect, positive patient attitude, and whether there is a national recommendation for the medication |
| Poulos 2013[[30](#_ENREF_30)]  USA and UK | Type 2 diabetes treatment | General practitioners and endocrinologists | 404; 204 US, 200 UK | 1. Glucose control within 3 months* 2. Frequency of injections* 3. Liver monitoring test* 4. Change in body weight* 5. Mild to moderate nausea* 6. Diarrhoea or vomiting* 7. Changes in the risk of a fata MI over 5 years* 8. Changes in depression symptoms* | GPs and endocrinologists in both countries had similar preferences. But in UK GPs had significant preferences for treatments that did not have liver monitoring requirements, whereas endocrinologists did not distinguish between treatments that did and did not have liver monitoring requirements.  Physicians were most sensitive to level of glucose control, % weight change associated with treatment, and reduction in risk of fatal MI associated with treatment |
| Riise 2016[[31](#_ENREF_31)]  Norway, Scotland, England | Medication prescribing | GPs | 907 | 1. Benefit/effect* 2. Patient cost* 3. Total cost* 4. Patient attitude* 5. Experience with medicine* | Physicians were most sensitive to effect size and patient preference of medicine. In Scotland and England physicians were more sensitive to patient preference for medicine compared with effectiveness, while as in Norway physicians were more sensitive to effectiveness |
| **Workplace characteristics** | | | | | |
| Ezatabadi 2016[[33](#_ENREF_33)]  Iran | Family contracts | General practitioners | 350 | 1. Contract duration* 2. Payment mechanism* 3. Contract employer* 4. Individuals covered* 5. Catchment area* 6. Right to provide* services outside package 7. Benefits* | Physicians were most sensitive to receiving benefits such as specialisation courses, as long as that was within 5 years being a family physician rather than 10 years. Physicians also sensitive to having the right to provide service outside of a specified package and the number of individuals covered |
| Gosden 2000[[34](#_ENREF_34)]  UK | Organisational characteristics in primary care | General practitioners who recently joined a new practice | 166 | 1. Out of hours work 2. Income* 3. List size* 4. Financial management 5. Opportunities to develop interests* 6. Size of team* 7. Daytime hours* 8. Deprivation of patient population* | The most important influence on physicians’ choice of practice was aversion to location in an area of high deprivation. Physicians were more likely to choose a practice that had an extended primary healthcare team, offered opportunities to develop specialist interests, offered higher income, shorter working hours, and smaller list sizes  WTP to avoid working in high depravation area: $5,029 per year |
| Holte 2015[[35](#_ENREF_35)]  Norway | Organisational characteristics in primary care | Final year medical students and interns | 831 | 1. Practice size* 2. Location* 3. Opportunity to control working hours* 4. Opportunity for professional development* 5. Income* | All of the attributes had a significant impact on choice of practice. A strong aversion was found for working in rural areas or in small practices. The respondents valued both the opportunity to control working hours and the opportunity for professional development. There was a high degree of variation in the preferences for all of the attributes except income. |
| Holte 2016[[36](#_ENREF_36)]  Norway | Organisational characteristics in primary care^[[3]](#footnote-3)^ | General practitioners | 1275 | 1. Type of practice* 2. Control of working hours* 3. Professional development* 4. Professional autonomy* 5. Income* | All of the attributes had a significant impact on choice of practice. GPs were most sensitive to change in income, followed by control over working hours and opportunities for professional development. GPs were more sensitive to loss of income than gain in income. |
| Li 2014[[37](#_ENREF_37)]  Australia | Rural medical workforce incentives | Rural GPs | 1720 | 1. Locum relief guarantee* 2. GP retention payment* 3. Rural skills loading* 4. Family isolation* | GPs were most sensitive to having a locum relief guarantee, GP retention payments, followed by rural skills loading. Family isolation payments only significantly influenced choosing rural practice if 100% costs were paid. When 50% of family costs were paid there was no significant impact on choice. |
| Pedersen 2012[[39](#_ENREF_39)]  Denmark | Organisational characteristics in primary care. | General practitioners | 1235 | 1. Number of GPs in practice* 2. Collaboration with other practices* 3. Working hours* 4. Yearly surplus* | All of the attributes had a significant impact on choice of practice. GPs had significantly different preferences depending on if they had solo or shared practices—GPs from solo preferences were significantly less likely to accept a new shared practice, had an aversion to hypothetical practices with more GPs, and were more responsive to changes in income than GPs in a shard practice. Both GPs in a solo practice and a shared practice were similarly averse to an increase in either administrative or patient related work. |
| Pedersen b 2012[[38](#_ENREF_38)]  Denmark | GP knowledge of patient preferences for organisational characteristics of general practice | Danish population and GPs | 698 Danish population; 969 GPs | 1. Telephone waiting time* 2. Opening hours* 3. Appointment* 4. Distance* 5. Waiting room* 6. Consultation time* 7. Who performs routine tasks* | GPs correctly predicted ranking of attributes for the patients but not size of effects, largely because GPs overestimated their own importance to the patients. |
| Pedersen 2014[[40](#_ENREF_40)]  Denmark | Organisational characteristics in primary care | GPs in training (in either shared or solo practices) | 485 | 1. Number of GPs in general practice* 2. Collaboration with other practices 3. Working hours* 4. Yearly surplus* | GPs in training prefer to work in smaller shared practices (2 GPs).  Willing to work in larger shared practices (with 3–4 GPs) if they receive an increase in income (~6,719 EUR per year)  Willing to take in more patient-related work (~26,875 EUR per year for 5 extra hours per week). |
| Scott 2001[[41](#_ENREF_41)]  UK | Organisational characteristics in primary care | GPs | 783 | 1. Opportunities for development 2. List size* 3. Daytime hours* 4. Income* 5. Admin time 6. Out of hours* 7. Use of guidelines* | Significant attributes included change in annual income, list size per GP, use of guidelines, daytime hours worked, out of hours work. Time spent on administration and ability to pursue special interests were not significant. |
| Scott 2013[[42](#_ENREF_42)]  UK | Organisational characteristics in primary care | GPs | 3727 | 1. Earnings* 2. Hours worked* 3. On-call arrangements* 4. Location* 5. Opportunities for social interaction* 6. Ease to arrange locum on short notice* 7. Team members* 8. Average consultation length* | Sixty five per cent of GPs chose to stay where they were in all choices presented to them.  Willingness to accept to move to inland town with less than 5000 population: ~$116,000 per year |
| Song 2015[[43](#_ENREF_43)]  China | Organisational characteristics in primary care | Primary care doctors and nurses | 282 doctors; 235 nurses | 1. Income* 2. Welfare benefits* 3. Essential equipment* 4. Career development* 5. Respect from the community* 6. Training opportunity* | Primary care providers were most sensitive to welfare benefits, having sufficient essential equipment, and respect from the community.  Doctors’ and nurses’ preferences over job attributes were similar.  Younger primary care providers were more likely to value training and career development opportunities. |
| Wordsworth 2004[[44](#_ENREF_44)] | Organisational characteristics in primary care | Principals and sessional GPs | 904 principals; 388 sessionals | 1. Consultation length* 2. Change in total hours worked per week* 3. Change in annual personal income* 4. Outside commitments* 5. Out-of-hours work* 6. Involvement in practice decisions* 7. Participation in CPD and training* | GPs were most sensitive to moving to medium or high intensity out-of-hours work. All GPS preferred longer consultations, no increase in working hours, and an increase in earnings.  Sessional GPs had significantly lower sensitivity to consultation length, hours of work, and a job offering enough CPD. |
| **Implementation and knowledge translation** | | | | | |
| Ammi 2016[[45](#_ENREF_45)]  France | Quality improvement programs | General practitioners | 303 | 1. Level of remuneration* 2. Method of remuneration* 3. Frequency of remuneration* 4. Prevention clinical guidelines * 5. Feedback on preventive practices 6. Continuing education in prevention 7. Type of practice 8. Assistance by non-physician providers during preventive work | Mixed logit demonstrates that heterogeneity in preferences is concentrated on the pay-for-performance component of the QIP, while the latent class model shows that physicians can be grouped in four homogeneous groups with specific preference patterns. Using policy simulation, we compare the French CAPI with other possible QIPs, and show that the majority of the physician subgroups modelled dislike the CAPI, while favouring a QIP using only non-financial interventions. |
| Chen 2010[[46](#_ENREF_46)]  Taiwan | Report card design for diabetes care | Hospital and primary care doctors | 221 | 1. Update frequency* 2. Risk adjustment* 3. Content information* 4. Display format* | Doctors’ preference attribute rankings were risk adjustment for patients, content information, display format and update frequency. One-year update frequency, risk adjustment, detailed scores of technical quality and interpersonal quality and bar chart display were the most important items noted in our survey. |
| Gong 2016[[47](#_ENREF_47)]  USA | Behavioural economics interventions to improve treatment of acute respiratory infections | Primary care physicians | 234 | 1. Suggested alternatives* 2. Accountable justifications* 3. Peer comparisons* 4. Pay-for-performance* 5. Additional time* | Prescribers overwhelmingly preferred suggested alternatives, followed by Peer comparison, then Accountable Justifications |
| Kjaer 2015[[48](#_ENREF_48)]  Denmark | Continuous professional development | General practitioners | 689 | 1. Appraisal assisted professional development planning* 2. Structure in programme* 3. Duration of program* 4. Course type 5. Type of educator* 6. Focus on exchange of experience* 7. Focus on implementation* | The attributes that had a significant impact on choice of professional development program included: partly centrally planned prof dev program, 10 day course (compared with either 5 or 15), GP trainers compared with specialist trainers, focus on exchange of experience, focus on implementation |
| Muller 2020[[50](#_ENREF_50)]  Germany | Adherence promoting programs | GPs, cardiologists, neurologists, ophthalmologists | 47 GPs, 175 specialists | 1. Validation status of the program* 2. Certification* 3. Type of intervention* 4. Time commitment* 5. Reimbursement* | Physicians were most sensitive to time commitment associated with the program and whether the program was validated.  Physicians older than 50 years had stronger preference for reimbursement compared with those younger than 50 years.  GPs had a greater preference for reimbursement compared with specialists. |
| Sicsic 2016[[49](#_ENREF_49)]  France | Financial and non-financial incentives in cancer screening | GPs | 402 | 1. Leaflet* 2. Training* 3. Listing* 4. Assistance* 5. Payment* | GPs are sensitive to both financial and non-financial incentives, such as a compensated training and systematic transmission of information about screened patients, aimed to facilitate communication between doctors and patients. There is also evidence that the level and variability of preferences differ across screening contexts, although the variations are not statistically significant on average. GPs appear to be relatively more sensitive to financial incentives for being involved in colorectal cancer screening, whereas they have higher and more heterogeneous preferences for non-financial incentives in breast and cervical cancers. |
| **Information systems and technology** | | | | | |
| Chudner 2019[[51](#_ENREF_51), [56](#_ENREF_56)]  Israel | Video consultations vs in-clinic consultations | Patients, physicians, policymakers | 508, 311, 141 | 1. Time till appointment* 2. Queuing time* 3. Relationship to physicians* 4. Quality of call* 5. Consultation purpose* 6. Patient self-management ability* | All attributes were significantly important in choosing videoconferencing vs in-clinic consultation for both the physician group. Physicians’ VC uptake was 30% in cases in which the consultation purpose was to diagnose and provide treatment and 48% in cases in which the consultation purpose was follow-up. |
| Wyatt 2009[[52](#_ENREF_52)]  UK | Information systems | GPs | 303 | 1. Speed of system response* 2. System reliability* 3. Users allowed access* 4. Place of access* | Doctors valued speed of response most in information systems but would be prepared to wait 28 seconds to access a system in exchange for improved reliability from 95% to 99%, a further 2 seconds for an improvement to 99.9% and 27 seconds for access to data from anywhere including their own home compared with one place in a single healthcare premises. However, they would require a system that was 14 seconds faster to compensate for allowing social care as well as National Health Service staff to read patient data. |

*P<0.05

1. “Some of the time” was not significant compared with “none of the time”. “A good bit of the time” and “all of the time” were both significant compared with “none of the time” [↑](#footnote-ref-1)
2. 5% risk of dry mouth was not significant compared with 0%. 10% and 33% risk were significant compared with 0%. [↑](#footnote-ref-2)
3. Data [↑](#footnote-ref-3)
